# High-resolution imaging of organic and inorganic nanoparticles at nanometre-scale resolution by X-ray ensemble diffraction microscopy

**DOI:** 10.1107/S1600577524010567

**Published:** 2025-01-01

**Authors:** Ning-Jung Chen, Chia-Hui Yeh, Huai-Yu Cao, Nai-Chi Chen, Chun-Jung Chen, Chun-Yu Chen, Yi-Wei Tsai, Jhih-Min Lin, Yu-Shan Huang, Chien-Nan Hsiao, Chien-Chun Chen

**Affiliations:** a Department of Engineering and System Science National Tsing Hua University Hsinchu30013 Taiwan; b Department of Physics National Tsing Hua University Hsinchu30013 Taiwan; c National Synchrotron Radiation Research Center Hsinchu30076 Taiwan; d Taiwan Instrument Research Institute, Hsinchu30076, Taiwan; RIKEN SPring-8 Center, Japan

**Keywords:** X-ray diffraction imaging, phase retrieval algorithms, X-ray optics, ensemble diffraction microscopy

## Abstract

Coherent diffraction microscopy (CDM) is a robust direct imaging method due to its unique 2D/3D phase retrieval capacity. Nonetheless, its resolution faces limitations due to a diminished signal-to-noise ratio (SNR) in high-frequency regions. Addressing this challenge, X-ray ensemble diffraction microscopy (XEDM) emerges as a viable solution, ensuring an adequate SNR in high-frequency regions and effectively surmounting resolution constraints. In this article, two experiments were conducted to underscore XEDM’s superior spatial resolution capabilities. These experiments employed 55 nm-sized silicon–gold nanoparticles (NPs) and 19 nm-sized nodavirus-like particles (NV-LPs) on the coherent X-ray scattering beamline of the Taiwan Photon Source. The core–shell density distribution of the silicon–gold NPs was successfully obtained with a radial resolution of 3.4 nm per pixel, while NV-LPs in solution were reconstructed at a radial resolution of 1.3 nm per pixel. The structural information was directly retrieved from the diffraction intensities without prior knowledge and was subsequently confirmed through transmission electron microscopy.

## Abstract 2

X-ray ensemble diffraction microscopy (XEDM) is an approach to enhance the signal-to-noise ratio in high-frequency regions of scattering. This article presents direct imaging of 55 nm core–shell silica–gold nanoparticles at a 6.8 nm resolution and 19 nm nodavirus-like-particles in solution at a 2.6 nm resolution to demonstrate the high spatial resolution that is difficult to achieve by conventional coherent diffraction microscopy.

## 1. Introduction

Obtaining a structure at nanometre resolution is a long-standing challenge in X-ray microscopy. X-ray coherent diffraction microscopy (XCDM) is one of the most promising methods to achieve high resolution since no lenses are needed to form an image, eliminating the need to consider aberration (Miao *et al.*, 1999 ▸; Neutze *et al.*, 2000 ▸; Robinson *et al.*, 2001 ▸; Miao *et al.*, 2002 ▸; Pfeifer *et al.*, 2006 ▸; Williams *et al.*, 2006 ▸; Thibault *et al.*, 2008 ▸; Tripathi *et al.*, 2011 ▸; Shapiro *et al.*, 2005 ▸; Song *et al.*, 2008 ▸; Nishino *et al.*, 2009 ▸; Giewekemeyer *et al.*, 2010 ▸; Chapman & Nugent, 2010 ▸). The resolution of XCDM is determined by the spatial frequency range, which is proportional to the scattering angle (Piggott, 1966 ▸). High incident flux and long acquisition times are typical approaches to obtaining valid signals at large scattering angles. While the brightness can be significantly enhanced by using an X-ray free-electron laser (XFEL) (Chapman, Barty, Bogan *et al.*, 2006 ▸; Seibert *et al.*, 2011 ▸; Clark *et al.*, 2013 ▸; Gallagher-Jones *et al.*, 2014 ▸; Kimura *et al.*, 2014 ▸; Ekeberg *et al.*, 2015 ▸; Hosseinizadeh *et al.*, 2017 ▸; Kurta *et al.*, 2017 ▸; Carlsten *et al.*, 2019 ▸), it is still challenging to achieve the desired resolution because of the radiation damage and poor scattering power of nano­materials. An alternative approach is to shorten the sample-to-detector distance. Researchers must insert a larger beam stop to prevent damage or saturation of the detector from the direct beam. When the area of missing data is larger than the central speckle of the diffraction pattern, the reconstruction is less reliable (Miao *et al.*, 2005 ▸). In addition to hardware enhancements, resolution can be improved by post-processing collected data. Recent progress shows that averaging resolution shells has the ability of enhancing the signal-to-noise ratio (SNR) in high-frequency regions, while deep learning also improves the quality of diffraction data, increasing the accuracy of phase recovery (Jung *et al.*, 2020 ▸; Lee *et al.*, 2021 ▸).

In our previous article, we proposed X-ray ensemble diffraction microscopy (XEDM) as a novel approach to overcome the above-mentioned difficulties. We showed its adaptability using both partially and totally coherent light sources (Chen *et al.*, 2023 ▸). Under the assumption of an ensemble containing large numbers of identical specimens with the same orientation and random distribution, the intensity profile of the diffraction pattern of the whole ensemble is approximated to the form factor of a single specimen multiplied by the number of identical specimens. Since a high number of specimens can greatly enhance the diffraction intensity, the effective signal can be extended to high-frequency regions. To demonstrate further that XEDM allows us to achieve a higher resolution than is available using conventional X-ray imaging techniques, we applied XEDM to abundant identical core–shell nanoparticles (NPs) with a diameter of around 55 nm. The experiment was conducted on the coherent scattering beamline 25A at the Taiwan Photon Source (TPS), with an estimated spatial resolution of about 3.99 nm from the cropped diffraction pattern. The results were analyzed through diffraction intensity estimation and comparison with images obtained using transmission electron microscopy (TEM), indicating high consistency.

Based on the successful results obtained from the experiment mentioned above, nodavirus-like-particles (NV-LPs) were used to validate the feasibility of XEDM across a biomacromolecule sample in solution. In conventional X-ray biological diffraction imaging, the main reasons for insufficient SNR in high-frequency regions are radiation damage and the poor scattering of biomacromolecules. We have demonstrated that XEDM can overcome these problems. Although the azimuthal angles of the patterns are averaged, only radial information is retained. However, we still achieved a reconstruction with a radial resolution of 1.3 nm per pixel, demonstrating the potential of XEDM. The reconstructed image was compared with cryo-EM images and X-ray crystallography data with rotation averaging, and it also showed a high consistency of the shell thickness.

## 2. Materials and methods for the experiment conducted with silica–gold core–shell NPs

### 2.1. Sample preparation of silica–gold core–shell NPs

The silica–gold NP solution (nanoComposix, San Diego, California, USA) was diluted to 1 ml in a microtube using ethanol at a concentration of 0.545 mg ml^−1^. Ultrasonic vibration (5 min) was applied to prevent the NPs from clustering. To lower the surface tension of the silicon nitride membrane window, the membrane was treated with acetone before depositing the samples. A small amount of the solution (10 µl) was dispersed onto a 100 nm-thick Si_3_N_4_ membrane (Norcada, Alberta, Canada) and finally air-dried. No additional alignment was needed because the core–shell nanoparticles were nearly identical and spherical under the current experimental setup. The membrane was mounted onto a sample holder. A snake-step scan with 10 µm per step was to be implemented using a piezo stage, so the scanning area was selected under an optical microscope before the experiment to ensure effective hits.

### 2.2. Experimental setup and image reconstruction of the silica–gold core–shell NPs

**Figure 1:**
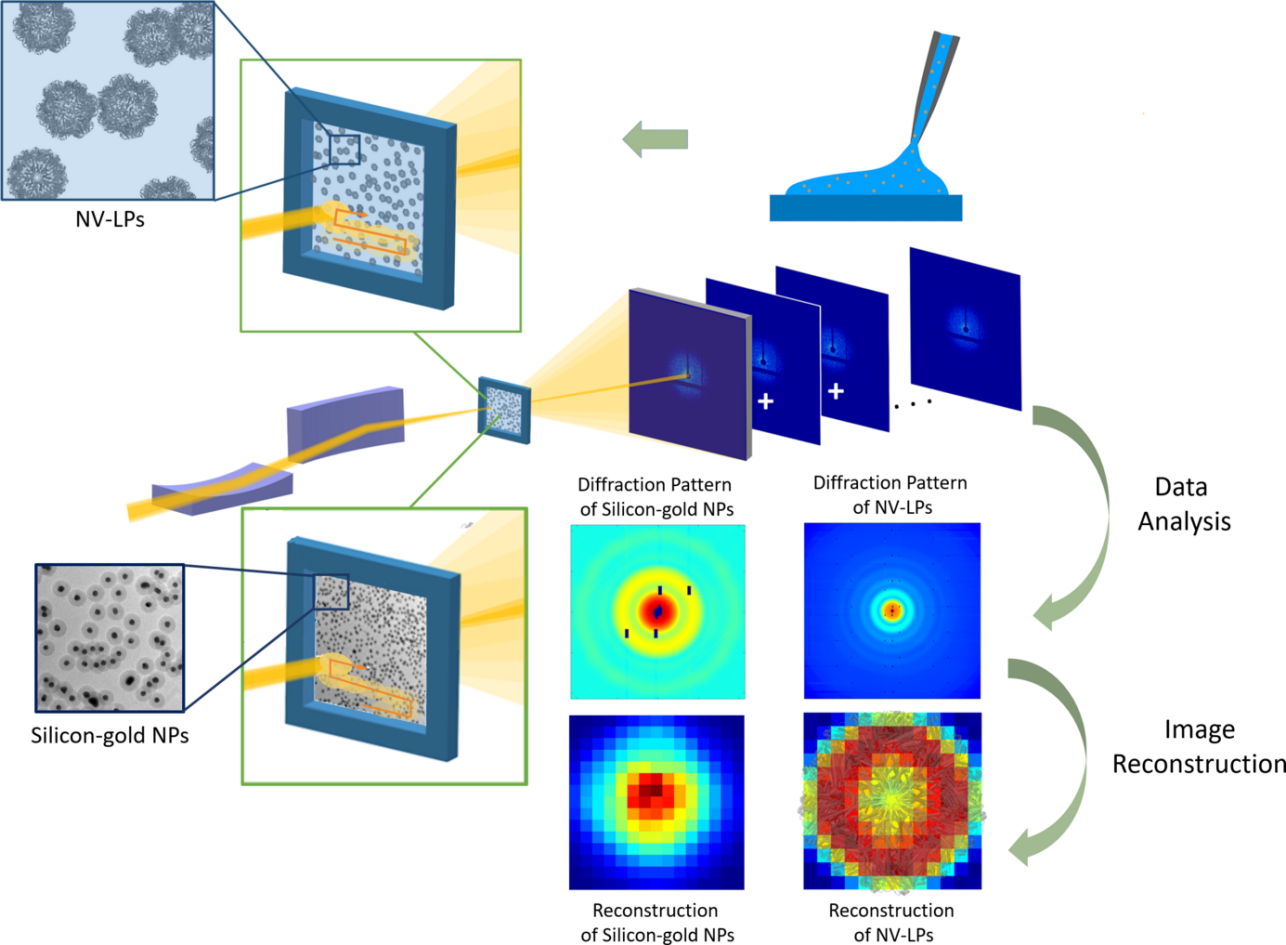
A schematic layout of the structural determination of organic and in­organic nanomaterials by XEDM experiment using third-generation synchrotron radiation. The incident beam performed snake-step scans across the specimens deposited on a silicon nitride membrane or in a silicon nitride envelope containing the solution. Multiple diffraction patterns were summed to generate a high-resolution diffraction pattern.

The experiment was conducted on TPS 25A with a coherent X-ray source (Fig. 1 ▸). The pulse energy of the X-ray beam was 8.821 keV to alleviate air scattering. The flux at the sample was measured as 1 × 10^9^ photons s^−1^ with a beam size of 6 µm × 18 µm. The exposures were automated using a 2D scan program that controlled both the time duration of the incidence and the sample motor stage. The detector was placed 2.08 m downstream of the sample and a circular central region with a diameter of 4.3 mm (∼57 pixels) was blocked by a beam stop. Before the beam stop was applied, an attenuator was inserted to locate the position of the incident beam on the detector. The beamline was operated under the top-up mode and the acquisition was automatically skipped when the electrons were injected. For each pattern, 60 s of data acquisition with a moving step size of 10 µm was employed. Finally, a total of 1676 diffraction patterns were recorded by an Eiger X 16M detector with a field of 4371 × 4150 pixels and a pixel size of 75 µm × 75 µm.

**Figure 2:**
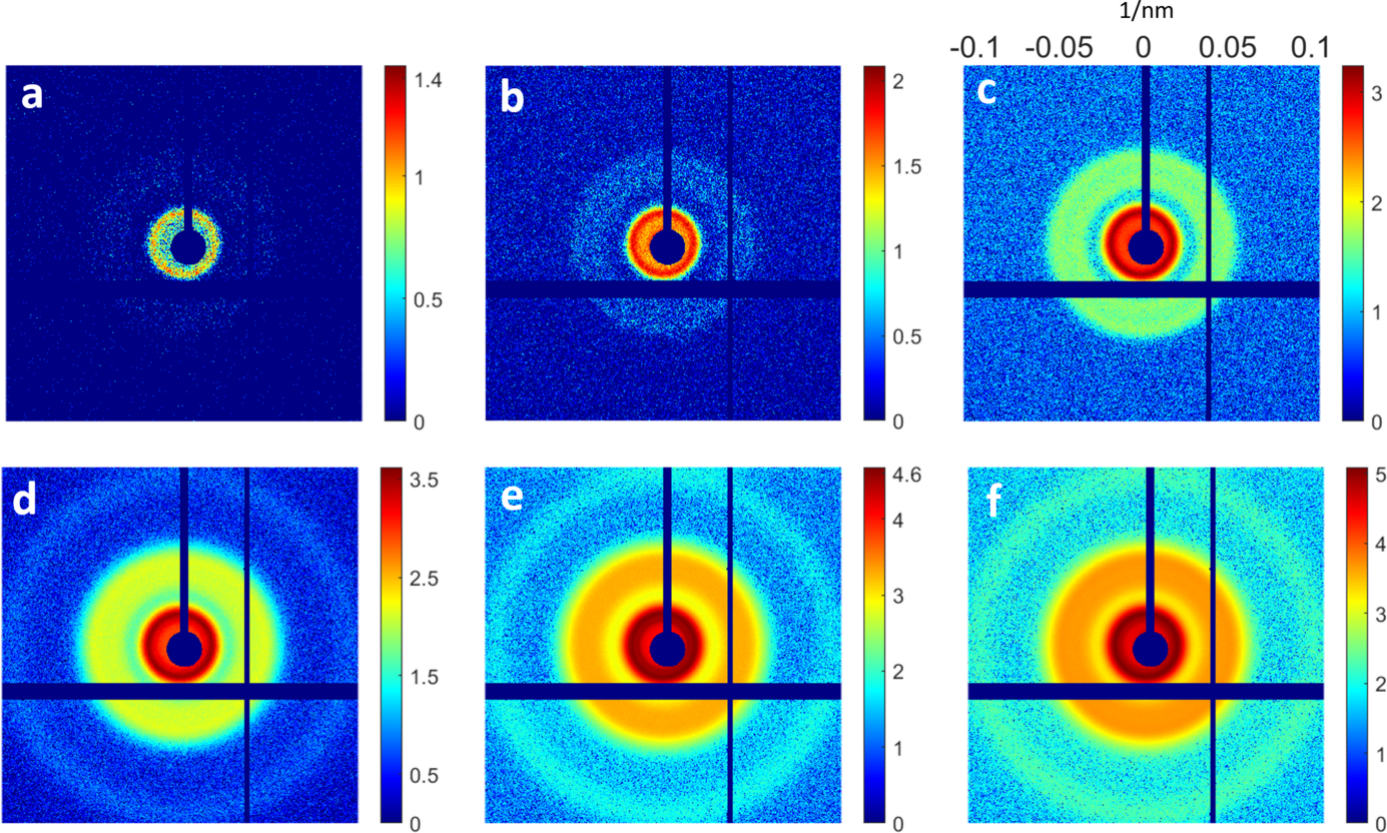
Summed patterns (on a logarithmic scale) for silica–gold NPs when different numbers of experimental diffraction patterns were applied, (*a*) 1, (*b*) 10, (*c*) 100, (*d*) 500, (*e*) 1000 and (*f*) 1676. Although the diffraction intensity is extremely weak in each pattern, the circular fringes can be easily recognized when summing a large number of diffraction patterns.

We summed all of the collected patterns, *i.e.* 1676 patterns (Fig. 2 ▸), to generate the final single diffraction image [Fig. 4(*a*)]. The experiment involved configuring the EIGER detectors with 16 small detectors, resulting in missing diffraction intensities in the gap areas between adjacent detectors and the beam-stop region.

Neglecting the absorption effect during scattering, the diffraction pattern was assumed to be symmetric (Vartanyants & Robinson, 2001 ▸). Several areas on two opposite sides of the approximate center were selected to find the accurate position of the origin, ensuring that no beam shift (compared to the measured position before the experiment) occurred during the data acquisition. After the origin of the diffraction pattern had been found, background subtraction was performed based on the ratio of frames. Pixels with no measured data or negative values were treated as missing data. The requirement of symmetry patched the missing data.

Considering the available range of the detector, 3×3 binning was performed to generate a final central symmetric diffractive amplitude. To minimize the effect of sample distribution while still enabling reconstruction, the central speckle (with a diameter of 151 pixels) was removed from the center of the diffraction pattern before phase retrieval. The reconstruction was performed using a guided hybrid input–output algorithm (GHIO) (Chen *et al.*, 2007 ▸) with 100 random initial phases and ten generations. The final reconstruction results must be determined based on the quality of each experimental data set, specifically the noise level. Therefore, in this experiment, the best 20% reconstructed images were averaged to generate the final reconstruction [Fig. 4(*b*)] (Chapman, Barty, Marchesini *et al.*, 2006 ▸; Yang *et al.*, 2019 ▸).

### 2.3. Results of the silica–gold core–shell NPs experiment

The length scale of our reconstructed result was verified by TEM images and the diffraction intensity calculation. From the TEM images, the diameters of the Au cores ranged from 16 nm to 21 nm and the diameters of the whole silica–gold NPs ranged from 53 nm to 58 nm. The averaged inner and outer diameters of the silica–gold NPs were measured as 19 and 55 nm, respectively. We then verified the experimental result through the simulation and compared it with the result from the TEM images. The number of NPs within the region of the incident beam can be roughly estimated from the TEM image at around 25280. We constructed silica–gold NP models of various sizes based on the experimental setup parameters, the atomic scattering factors of silica–gold NPs and the number of NPs within the beam. Subsequently, we calculated the number of photons recorded at each detector pixel (Glatter *et al.*, 1982 ▸; Lan *et al.*, 2014 ▸) and performed an error analysis with the experimental results to confirm the consistency between the experimental results and TEM images.

The diffraction intensities were estimated to verify the agreement between theory and experiment. The theoretical scattering intensity was quantified as
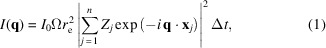
where *I*_0_ is the incident X-ray flux on the sample per unit area per unit time, Ω is the solid angle of the detector pixel, *r*_e_ is the classical radius of the electron, *Z*_*j*_ and **x**_*j*_ are the atomic number and the coordinate of the *j*th atom, respectively, Δ*t* is the time duration of each pattern, and the scattering vector **q** = **k**_*j*_ − **k**_*i*_ is the difference between the scattered and initial wavevectors.

**Figure 3:**
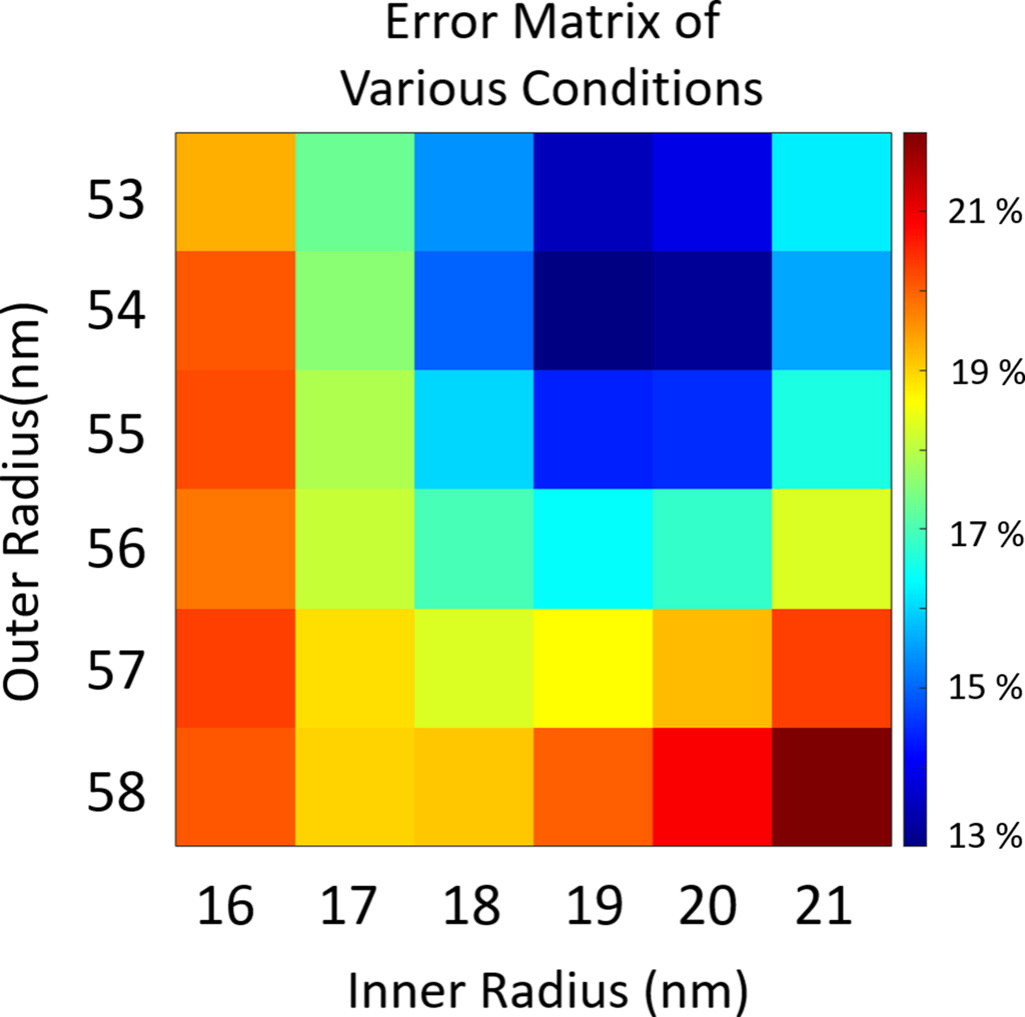
An error matrix of the experimental results for the silica–gold NPs compared with the model under various conditions, presented using a 2D color map. The calculated diffraction patterns of silica–gold NP models with inner (from 16 nm to 21 nm) and outer (from 53 nm to 58 nm) diameters were compared with the recorded diffraction pattern. The error matrix shows that the experimental inner and outer diameters of the silica–gold NPs were determined to be 19 nm and 54 nm, respectively, with a minimum error value of 13%.

Various inner and outer diameters of the silica–gold NP model were used to simulate a single NP’s diffraction amplitude (form factor). A multiplier (6320) determined from the number of NPs within the beam size was used to amplify the diffraction amplitude. The central speckle was removed before calculating the error to alleviate the effect of the distribution of NPs. We scanned possible inner and outer diameters ranging from 16 to 21 nm and 53 to 58 nm, respectively, and then compared them (Fig. 3 ▸) with the recorded diffraction patterns using the error metric
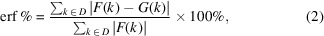
where *F*(*k*) are the Fourier amplitudes of our experiment result, and *G*(*k*) are the Fourier amplitudes of the simulated model with corresponding inner and outer diameters. *D* denotes the region of valid diffraction data.

**Figure 4:**
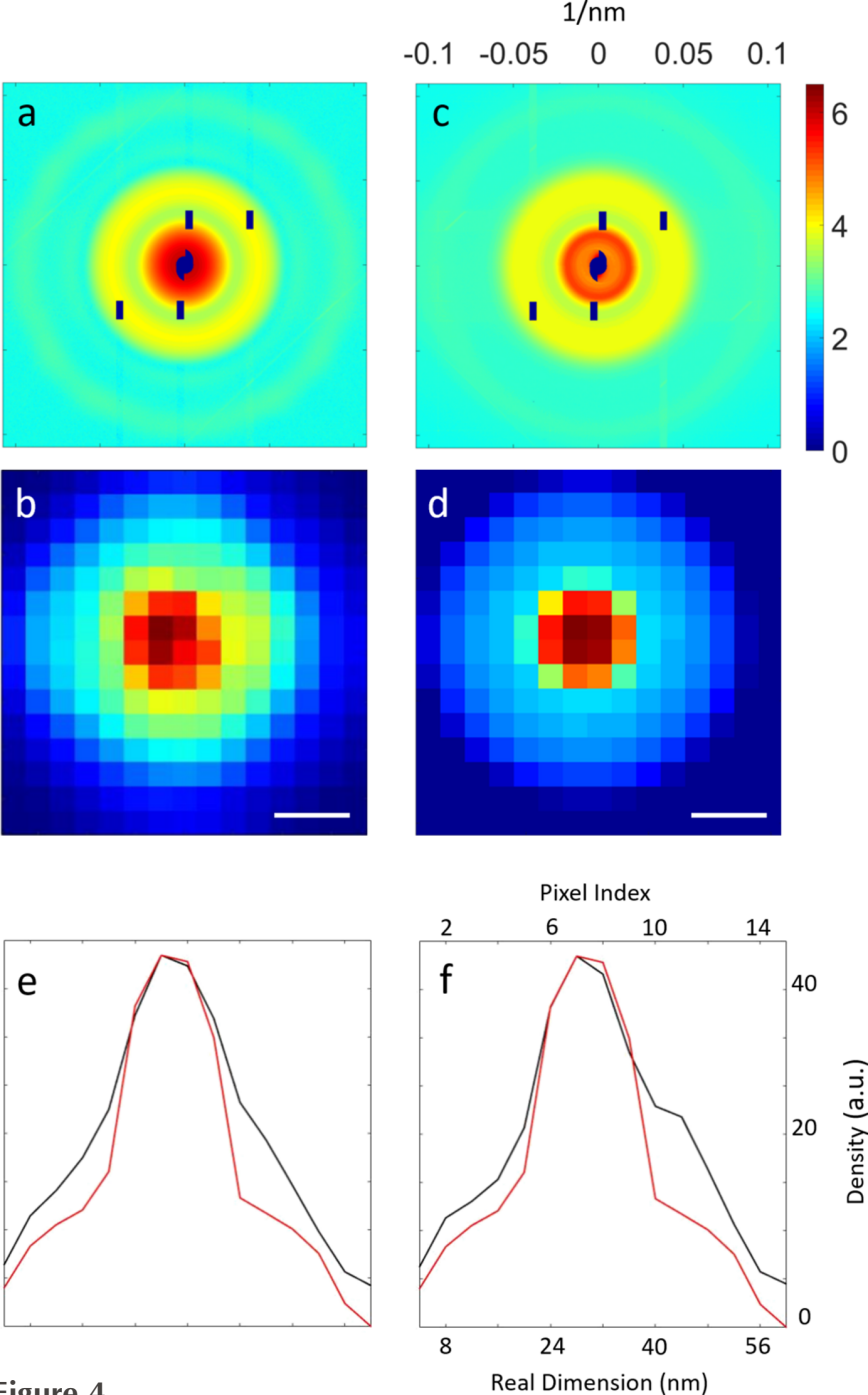
Structural determination and intensity estimation of the silica-coated gold NPs used in the proof-of-principle experiment. (*a*) The analyzed final diffraction amplitudes from the experiment on a logarithmic scale and (*b*) the reconstruction by the guided hybrid input–output method at a radial resolution of 3.99 nm per pixel with a scale bar of 10 nm. (*c*) The simulated diffraction amplitudes on a logarithmic scale from the 25280 silica–gold NPs and (*d*) the silica–gold NP model at the same radial resolution per pixel and scale bar as in panel (*b*). The inner and outer diameters of the silica–gold model were 19 and 54 nm, respectively. (*e*) The line profiles of the central columns in panels (*b*) and (*d*), depicted in black and red, respectively. (*f*) The line profiles of the central rows in panels (*b*) and (*d*), depicted in black and red, respectively.

From Fig. 3 ▸, the minimum of the error metric was 13% when using 19 and 54 nm for the model. The accuracy of the consistency is also demonstrated by Figs. 4 ▸(*e*) and 4 ▸(*f*), showing the line profiles for the central column and row, respectively, of the reconstructed image [Fig. 4 ▸(*b*)] and the simulated model [Fig. 4 ▸(*d*)]. Despite a 1 nm difference in the outer diameter between our results and the TEM images, considering the differences in radial resolution per pixel of the reconstructed images (±3.99 nm) our reconstructed results still show a high level of agreement with the TEM images.

**Figure 5:**
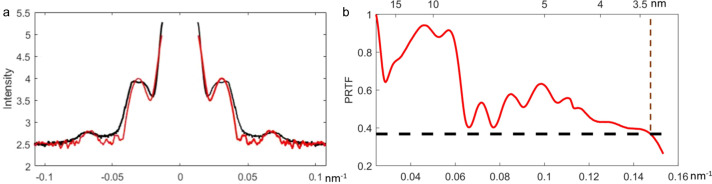
(*a*) The diagonal line profiles of the experimental intensity (black) and simulated intensity (red) for silica–gold NPs. (*b*) The phase-retrieval transfer function calculated from 20 independently reconstructed images. Based on the criterion of 1/*e*, the half-period radial resolution of the reconstruction is estimated to be 3.4 nm.

Given that the inner and outer diameters of the core–shell silica–gold NPs are 19 nm and 54 nm, respectively, the number of scattered photons from a single silica–gold NP at each pixel can be calculated based on the experimental setup. A number was needed to magnify the calculated pattern to match the experimental pattern. We found that using a magnitude of 25280 (the estimated number for the total sample within the beam in each incidence) together with the background pattern generated an excellent fit for the experimental measurements. Fig. 5 ▸(*a*) presents an excellent agreement between the simulated diffracted intensity [Fig. 4 ▸(*c*)] and the measured intensity [Fig. 4 ▸(*a*)].

The images were also used to calculate the phase-retrieval transfer function (PRTF) (Chapman, Barty, Marchesini *et al.*, 2006 ▸). The PRTF is a reliable method for resolution estimation in the field of XCDM with the definition 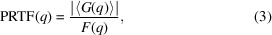
where *G*(*q*) and *F*(*q*) are the reconstructed and measured amplitudes, respectively. We selected the 20 best reconstructed images of the silica–gold NPs to calculate the PRTF curve. With a cutoff of 1/*e*, the half-period (*i.e.* pixel) radial resolution is determined to be approximately 3.4 nm [Fig. 5 ▸(*b*)].

To quantify more accurately the differences between the reconstructed images and the model, the structural similarity index measure (SSIM) was introduced (Wang *et al.*, 2004 ▸). The SSIM assesses perceptual similarity by considering contrast, structure and luminance. After calculation, the SSIM between our reconstructed result and the model was 0.8858, indicating a high level of consistency. The slight differences between the reconstruction and the model are due to variations in the sizes of the silica–gold NPs (inner and outer diameters ranging from 16 to 21 nm and 53 to 58 nm, respectively), as shown in the TEM images. If the particles in the space are more nearly identical, the difference between the reconstructed result and the model will be smaller. The boundary between the core and the shell will also become more distinct.

## 3. Materials and methods for the experiment conducted with NV-LPs

### 3.1. Sample preparation from PvNV capsid protein and *T* = 1 PvNVSd virus-like particle assembly.

The NV-LPs used in this research were analyzed at the NSRRC-NCKU Protein Crystallography Laboratory (Chen *et al.*, 2019 ▸; Chen *et al.*, 2015 ▸). Protein expression in *Escherichia coli* host strain BL21-DE3, purification and capsid assembly *in vitro* were characterized as previously described using an *N*-terminal hexahistidine-SUMO-tagged construct that produces the ΔN-ARM-PvNVSd (encoding residues 38–250), in which PvNV capsid protein (GenBank accession No. ABO33432.2) is missing the first 37 residues and residues 251–368. An enclosure was necessary to carry out the XEDM experiment on fully hydrated biological specimens. Approximately 10 µl of capsid protein was enclosed between two square silicon nitride membrane windows and sealed with high-vacuum grease.

### 3.2. Experimental setup and data processing of the NV-LPs experiment

These NV-LPs were assumed to be deposited randomly on the silicon nitride membrane windows. The samples were mounted in air on a three-axis (*XYZ*) piezo nanopositioning stage. All experimental parameters were the same as in the previous experiment conducted with silica–gold core–shell NPs, except the beam size was measured as 5.5 µm × 8.9 µm and the acquisition time was adjusted to 1 s per pattern to reduce the radiation damage to the biomolecules (Fig. 1 ▸). Considering the size of the beam, the number of NV-LPs within the beam is estimated to be around 6.7122 × 10^9^.

**Figure 6:**
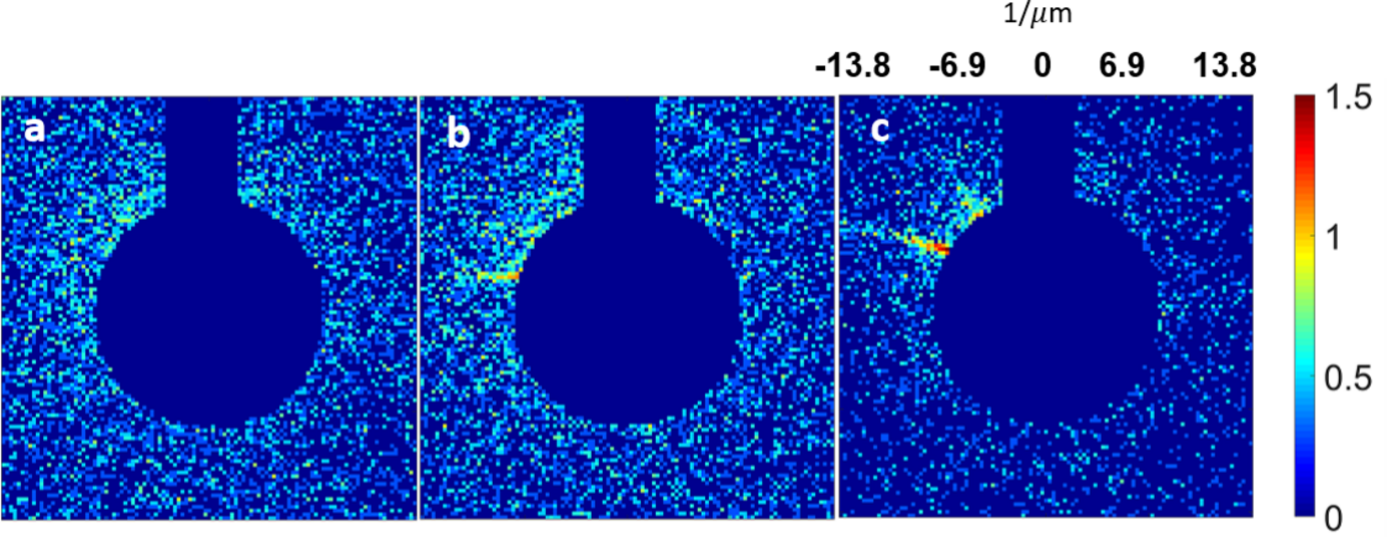
Three diffraction patterns from the NV-LP solution with different χ^2^ values, (*a*) 507.04, (*b*) 806.34 and (*c*) 2039.76. Since streaks should not appear in the diffraction patterns, we removed those diffraction patterns with a χ^2^ value larger than 800. The color bar is on a logarithmic scale.

Based on the aforementioned data-acquisition scheme, snake-like scanning was applied to most of the silicon nitride membrane envelope areas where no prior knowledge of the sample was available, and we collected 85616 diffraction patterns from NV-LPs. Considering that some incidences hit the silicon frame rather than the silicon nitride membrane, we first examined the total intensity of each pattern. Here, 5035 patterns were removed because the total intensity was ten times smaller than other patterns. Some of the remaining 80581 patterns contained edge scattering from the silicon frame or dust. A χ^2^ value as defined below was used to monitor the pattern when edge scattering along a specific direction occurred (Cochran, 1952 ▸):
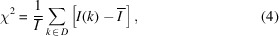
where *I*(*k*) and 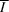 are the intensity on individual pixels and the averaged intensity of selected pixels, respectively. *D* denotes the region of selected diffraction data. In our data analysis, *D* formed a circle including all the pixels next to the beam stop. Typically, χ^2^ was approximately 500. Then, 3070 patterns with significantly large χ^2^ values (>800) were further removed. Fig. 6 ▸ shows the χ^2^ values for various patterns. Finally, the scattering signal from the specimens was expected to be stronger than the background scattering. The total intensity examination was again imposed on the remaining patterns. A total of 341 out of 77511 patterns with weaker total intensity were manually checked and summed as the background pattern.

**Figure 7:**
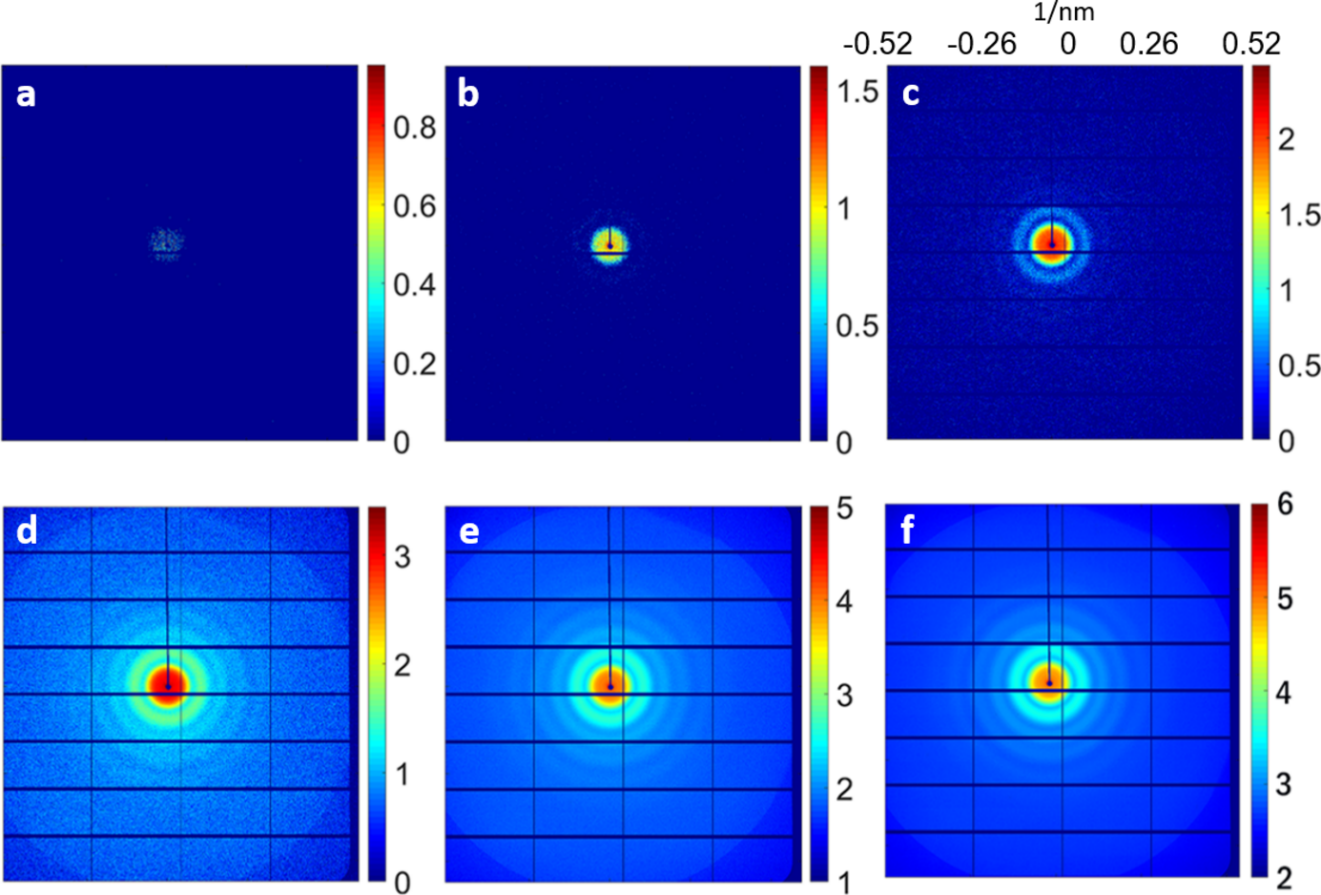
Summed patterns (on a logarithmic scale) when different numbers of experimental diffraction patterns were applied for the NV-LP solution, (*a*) 1, (*b*) 10, (*c*) 100, (*d*) 1000 and (*e*) 10000. Panel (*f*) shows that the scattering signal from the specimens was expected to be stronger than the background scattering after summing 77170 diffraction patterns.

Fig. 7 ▸ shows different accumulations of the diffraction patterns. The signal of the high-frequency region becomes recognizable when summing a large number of diffraction patterns [Figs. 7 ▸(*b*), 7 ▸(*c*), 7 ▸(*d*) and 7 ▸(*e*)]. Lastly, the final diffraction amplitude with an accumulated 77170 diffraction patterns was obtained [Fig.7(*f*)]. The final reconstruction was also generated using the GHIO algorithm with the same procedure.

### 3.3. Results of the NV-LPs experiment

**Figure 8:**
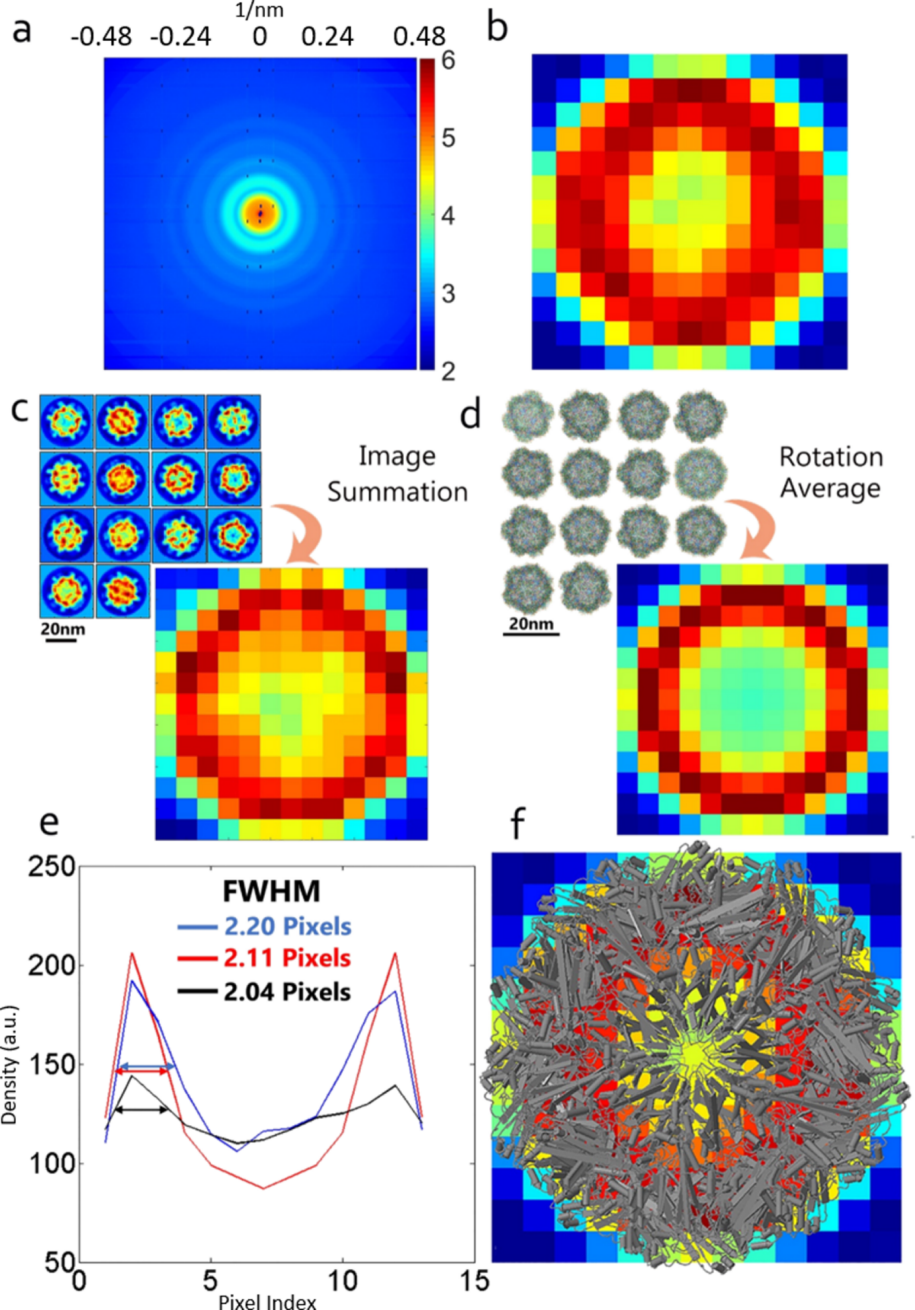
The calibration of the reconstructed NV-LP sample at a radial resolution of 1.3 nm per pixel. (*a*) The analyzed final diffraction pattern from a total of 85616 patterns and (*b*) the image retrieved by the guided hybrid input–output method. (*c*) An approximate rotation-averaged image of nodavirus generated by adding together cryo-EM images of 14 classifications. (*d*) A rotation-averaged image of the NV-LP model generated by summing all possible orientations with 1° increments. (*e*) A line-profile comparison of the central columns in panels (*b*)–(*d*). The full width at half maximum (labeled by double arrows) of the sharpest profile demonstrates a high consistency of the shell thickness across currently existing approaches: crystallography (red), cryo-EM (black) and our approaches (blue). (*f*) An overlay of the overall protein structure and the reconstructed image on the same scale depicts the density distribution of the hollow structure observed in both cases.

We identified the density distribution in the reconstruction using two currently available approaches. First, a measurement of the dimensions of the core–shell structure was performed from the image acquired by cryogenic electron microscopy (cryo-EM) (Chen *et al.*, 2019 ▸). The cryo-EM images of nodavirus with 14 classifications were added together to produce an approximate rotation-averaged image with a radial resolution of 1.3 nm per pixel [Fig. 8 ▸(*c*)]. Second, the atomic coordinates of NV-LP in the Protein Data Bank (PDB) were employed to allocate a 3D image with a voxel of 1.3 nm × 1.3 nm × 1.3 nm. The projective NV-LP image was generated by averaging the 3D NV-LP model projected along all possible orientations with 1° increments [Fig. 8 ▸(*d*)]. The line profiles of the reconstructed image, the cryo-EM image and the allocated projection demonstrate that the sharpest shell thickness from different identification methods was consistent at ∼2.6 nm (∼2 pixels); therefore, a radial resolution of 1.3 nm per pixel was achieved [Fig. 8 ▸(*e*)]. An overlay of the overall protein structure and the reconstructed image on the same scale verified the morphology of the hollow structure [Fig. 8 ▸(*f*)]. Compared with the rotation-averaged image of the NV-LP model, the error in the reconstructed image is 15.7%.

Since the effects of ice and solvent must be considered in the cryo-EM image and our reconstruction, the shell thickness is a good indicator of the structural determination. Although only the NV-LP model produces a symmetrical projected image, the shell thickness can still be identified by comparing the full width at half maximum of the sharpest profile. The representative line profiles of the central columns of the cryo-EM image, allocated image and raw reconstruction confirm the accuracy of the thickness determination of the shell structure.

## 4. Discussion and conclusions

In summary, instead of imaging a single particle with a considerable dose, we have utilized the common identity of specimens to overcome the bottleneck of the current resolution limit caused by radiation damage. XEDM has demonstrated its capability of addressing the issue of insufficient high-frequency signal and has shown an impressive radial resolution of 3.4 nm per pixel using identical 55 nm core–shell NPs and a radial resolution of 1.3 nm per pixel using identical 19 nm NV-LPs on the coherent scattering beamline TPS 25A, surpassing other X-ray imaging techniques.

For samples lacking specific alignment treatment, XEDM is commonly compared with small-angle X-ray scattering (SAXS) (Glatter *et al.*, 1982 ▸). SAXS, analyzed through the radial distribution function (RDF) from data collected by a 1D/2D detector (Dinnebier & Billinge, 2008 ▸), is a well established method for unveiling the 3D density variation of a reference particle based on a predetermined model and the data fitting of the measured RDF curve. However, SAXS faces severe low-resolution and ill-information issues, and its solution is not uniquely defined in principle. While some research suggests that *ab initio* structural determination can be achieved solely through SAXS data (Grant, 2018 ▸), it has been noted that this approach is less universally applicable (Konarev & Svergun, 2021 ▸). In cases where samples are not aligned, the obtained scattering information is essentially equivalent to 2D SAXS. XEDM offers an alternative approach by directly imaging the 2D projection of the specimen without ambiguity, achieved through unique phase retrieval from the diffraction intensities alone.

Hence, the advantages of XEDM are fourfold. First, the signal of high-angle scattering can still be acquired by accumulating subsequent incidences at different positions, even if specimens were destroyed in the previous incidence. Second, the SNR of diffraction intensity obtained from multiple specimens, as opposed to a single specimen, is significantly improved. Specimens outside the temporal coherence length also positively contribute to the form factor of the specimen. Third, the low-resolution area is eliminated to mitigate the impact of particle distribution before initiating the image reconstruction. As a result, the size of the missing center becomes less of a concern in the reconstruction process. Fourth, model-free structural determination is carried out without ambiguity, as 2D sampling proves to be a more general approach than 1D sampling.

XEDM represents a breakthrough in surpassing the limits of pixel resolution, and this approach can be applied across various types of light sources, such as partial/coherent X-ray or even electron sources, based on different types of samples. While applying to X-ray imaging, XEDM expands the range of sample choices, such as liquid biological samples, and effectively addresses beam shift issues arising from environmental factors during experiments. According to theory, the scattering intensity of an ensemble containing *N* particles is proportional to *N*^2^ when the coherent length of the light source is larger than the entire ensemble. However, if the coherent length is greater than the size of individual particles but smaller than the entire ensemble, the scattering intensity becomes proportional to *N*. In the current demonstration, a third-generation synchrotron light source was utilized. A coherent light source was achieved by inserting slits to limit the beam size, thereby enhancing spatial coherence. However, this approach results in a significant loss of flux compared to a partially coherent light source (*i.e.* the light source mentioned in the second condition described above), suggesting that the trade-off in flux for increased coherence may not offer a substantial advantage. If the selected light source does not require slits to achieve fully coherent conditions, such as in the case of an XFEL, then it would be possible to amplify high-*Q* signals to a greater extent, as previously discussed.

The success of the two experiments conducted in this article can be attributed to using spherical (or near-spherical) samples. For more anisotropic specimens some alignment is necessary. The current experiments aimed to demonstrate that XEDM can improve the resolution of X-ray imaging techniques. The next challenge for XEDM lies in effectively and accurately controlling the orientation of samples, especially for biological macromolecules. With a suitable alignment of specimens, we believe the resolution can be improved to the atomic scale by combining XFELs with a shorter distance. Compared with crystallography and cryo-EM, this is not only an alternative method for visualizing the structures of macromolecules but also a unique approach to imaging macromolecules in solution. We anticipate this work will significantly inspire SAXS, XCDI, XFEL science and structural biology researchers.
